# Species conservation profile and revision of *Rhinolophus acuminatus* (Chiroptera, Rhinolophidae) from Southeast Asia

**DOI:** 10.3897/BDJ.13.e162374

**Published:** 2025-11-28

**Authors:** Nazifah Fitriyah Zariman, Juliana Senawi

**Affiliations:** 1 1Department of Biological Sciences and Biotechnology, Faculty of Science and Technology, Universiti Kebangsaan Malaysia, 43600, Bangi, Malaysia 1Department of Biological Sciences and Biotechnology, Faculty of Science and Technology, Universiti Kebangsaan Malaysia, 43600 Bangi Malaysia; 2 2Langkawi Research Centre, Tuanku Abdul Halim Mu’adzam Shah Campus, Universiti Kebangsaan Malaysia, Teluk Yu Road, Burau Bay, 07100, Langkawi, Malaysia 2Langkawi Research Centre, Tuanku Abdul Halim Mu’adzam Shah Campus, Universiti Kebangsaan Malaysia, Teluk Yu Road, Burau Bay, 07100 Langkawi Malaysia

**Keywords:** Area of Occupancy, bat conservation, Extent of Occurrence, IUCN Red List Assessment

## Abstract

**Background:**

*Rhinolophus acuminatus* was first evaluated for its conservation status in 1996, with subsequent assessments conducted in 2008 and most recently in 2019, during which it was categorised as Least Concern. These evaluations, however, were largely based on limited occurrence records and a general list of countries where the species was known to occur. Recent discoveries have documented new distribution records, indicating a broader geographic range than previously recognised. Additionally, the availability of a more precise locality data has contributed to a more comprehensive understanding of the species distribution and ecological context.

**New information:**

This study provides novel insights into the distribution of *Rhinolophus acuminatus*, including newly-documented localities, an updated elevational range and refined regional records. Notably, Sarawak (Malaysian Borneo) and Brunei were not previously recognised as part of the species’ range during its most recent IUCN assessment. We report the first confirmed occurrence of *R. acuminatus* in Sarawak, specifically within Gunung Mulu National Park, a UNESCO World Heritage Site, known for its exceptional biodiversity and extensive limestone karst systems. Additionally, a new locality record from Melilas in Brunei further expands the species’ known distribution on the island of Borneo. The record from Sarawak represents a newly-documented extant range within Malaysian Borneo, while the Brunei record constitutes a new country record for *R. acuminatus*. This study also presents the most comprehensive and detailed locality data for the species since its last detailed assessment in 2019. The species’ elevational range has been revised from the previously reported maximum of 1,676 m above sea level (a.s.l.) in the 2019 IUCN assessment to a new upper limit of 3,943 m a.s.l., based on records from Mount Kinabalu in Sabah, Malaysian Borneo, indicating the species' presence in upper montane forest habitats. Furthermore, this study provides the first quantitative estimates of the species’ Extent of Occurrence (EOO), calculated at 6,957,361.5 km², Area of Occupancy (AOO) at 608 km² and 149 number of locations. EOO mapping further illustrates the species’ broad distribution across the Southeast Asian region. Collectively, these findings offer critical data for future reassessments of the conservation status of *R. acuminatus* under the IUCN Red List criteria.

## Introduction

Currently, a total of 1,500 bat species are recognised globally (Simmons and Cirranello 2025). The family *Rhinolophidae* Gray, 1825 includes only a single genus, *Rhinolophus* Lacepède, 1799 (Csorba et al. 2003). This genus is restricted to the Palaeotropical Region and is believed to have originated in the Oriental biogeographic zone (Chornelia and Hughes 2022). Species within *Rhinolophus* are easily distinguished by their complex noseleaf structure, which includes a raised sella, a connecting process and a horseshoe-shaped structure covering the nostrils and upper lip (Csorba et al. 2003). This specialised morphology is thought to play a vital role in echolocation, enabling these bats to navigate and forage effectively in complete darkness (Curtis and Simmons 2017, Pavey 2021). The high diversity of *Rhinolophus* in Southeast Asia highlights the region’s importance for bat conservation (Kingston 2010).

The genus was first divided into morphological groups by Knud Andersen in 1918, based primarily on the structure of the noseleaf. The pusillus group contains 11 recognised species that share a triangular-pointed shape of the connecting process (Andersen 1918, Csorba et al. 2003, Bates et al. 2004). Within this group, *Rhinolophus acuminatus* is notably larger than other members (Bates et al. 2004, Francis 2019). Although it closely resembles *R. affinis* in overall morphology, it can be distinguished by the shape of the connecting process, which is triangular in *R. acuminatus* and rounded in *R. affinis* (Francis 2019). Despite being placed in the same morphological group as *R. pusillus*, phylogenetic evidence indicates that *R. acuminatus* belongs to a different evolutionary lineage (Csorba et al. 2003).

Ecological studies have revealed variations in both body size and roosting behaviour across different regions. In Myanmar, males are generally larger than females (Bates et al. 2004). Observations from the same region recorded colonies of around 100 individuals, while surveys in Vietnam found aggregations of up to 500 bats (Bates et al. 2004, Thong et al. 2019). In contrast, studies in Malaysia documented this species roosting solitarily or in pairs (Kingston et al. 2009).

*Rhinolophus acuminatus* is distributed widely across Southeast Asia. Its most recent IUCN Red List assessment in 2019 classified the species as Least Concern (Thong et al. 2019). However, the assessment lacked detailed information, particularly regarding range metrics, such as the Extent of Occurrence, Area of Occupancy and the number of locations. Since then, expanded research efforts have led to new locality records and improved knowledge of the species’ elevational range. These findings reveal significant gaps in the previous assessment and underscore the need for updated data to better understand the species’ distribution, ecological requirements and potential threats. Including such information will enhance the accuracy of conservation assessments and provide information for more effective management strategies.

The objective of this study is to provide updated and comprehensive data to support the reassessment of the IUCN conservation status of *R. acuminatus*, with a particular focus on its distribution, elevational range and key conservation metrics.

## Material and Methods

Locality data for *Rhinolophus acuminatus* were compiled from a total of 153 sites, comprising 148 sites from published records and five sites from field surveys, conducted for the present study. Field surveys were conducted primarily in Malaysia using various bat-trapping techniques, including harp traps, mist nets and high nets. Harp traps were strategically placed across trails, while mist and high nets were deployed in open areas, such as above rivers, forest gaps and clearings within the study sites. Traps and nets were operated from dusk (1900 hrs) to dawn (0700 hrs). The geographic coordinates of each trapping site were recorded using a Garmin GPSMAP 65s device. Morphological measurements were taken from each captured individual, including forearm length, body weight, sex, age and reproductive status. Species identification was conducted using the taxonomic key for Peninsular Malaysian bats, as described by Kingston et al. (2009) and Borneo Malaysia bats, as described by Yasuma et al. (2005a) and Yasuma et al. (2005b).

Additional locality data were obtained from the Zoological Museum of Universiti Kebangsaan Malaysia (UKM), where both skull and wet specimens of *R. acuminatus* were examined and corresponding locality information was recorded. All geographic coordinates derived from literature, field surveys and museum records were standardised and converted into decimal degree format.

**Figure 1.**
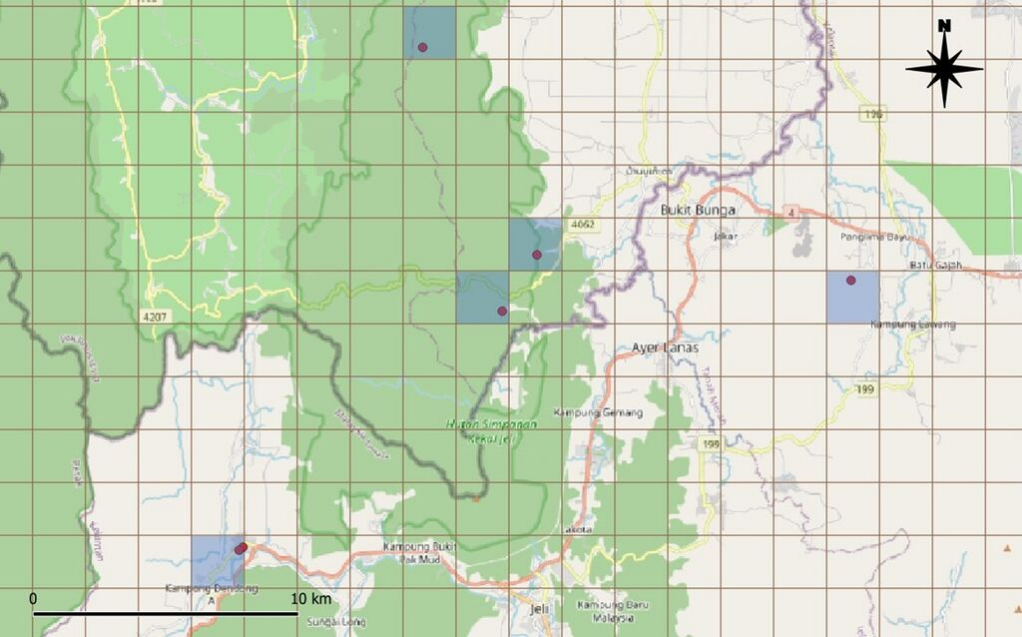
Illustration of scale dependence in the calculation of Area of Occupancy (AOO). The red dots represent six locality points, distributed across five occupied grid cells (5 locations). Based on the standard 2 km × 2 km grid, the estimated AOO for this area is 5 × 4 km² = 20 km².

A distribution map was created using QGIS version 3.34.1, incorporating spatial data for each country sourced from GADM version 4.1 (2025). All maps were set to the WGS 1984 coordinate reference system (EPSG:4326). The Area of Occupancy (AOO) was calculated using the 'Grid' tool in QGIS to create a 2 km x 2 km grid around each locality point, following IUCN guidelines (IUCN Standards and Petitions Committee 2022). The AOO estimates were obtained by counting the number of occupied cells times the area of an individual cell (4 km^2^). Detailed protocols for locality data collection, AOO and EOO calculations are published and accessible at protocols.io (Zariman and Senawi 2025) (Fig. 1).

The Extent of Occurrence (EOO) was estimated using the IUCN EOO Calculator Toolbox in ArcMap version 10.8.1. Prior to running the toolbox, presence, origin and seasonal attributes were assigned to each locality point. EOO is defined as the area within the smallest continuous imaginary boundary that encompasses all known, inferred or projected sites of a taxon’s current presence, excluding instances of vagrancy. It is important to note that EOO is not intended to reflect the total area of occupied or suitable habitat, nor is it a general representation of the species’ overall range. EOO is typically calculated using a minimum convex polygon, which is the smallest polygon in which all internal angles are less than or equal to 180 degrees and which contains all occurrence points.

The number of locations was determined using the 'Google Satellite Image' function in QGIS, guided by the IUCN Red List definition of a “location” as a geographically or ecologically distinct area in which a single threatening event can rapidly impact all individuals present. This definition differs from the broader terms “location” or “locality” commonly used in biogeographic studies. In the assessment of *R. acuminatus*, all known occurrences were considered, regardless of current threat levels. The primary plausible threat to the species is habitat loss due to deforestation, which affects both roosting and foraging habitats. According to IUCN Red List criteria, if multiple subpopulations occur within an area that could be affected by a single threatening event, they are counted as one location. Conversely, if a subpopulation spans an area larger than could be impacted by a single event, it may be counted as multiple locations (IUCN Standards and Petitions Committee 2022).

## Species Conservation Profiles

### Rhinolophus acuminatus

#### Species information

Scientific name: Rhinolophus acuminatus

Species authority: Peters, 1871

Common names: Acuminate Horseshoe Bat (English), Kelawar Ladam Kenarong (Malay).

Kingdom: Animalia

Phylum: Chordata

Class: Mammalia

Order: Chiroptera

Family: Rhinolophidae

Taxonomic notes:

*Rhinolophus acuminatus* Peters, 1871 belongs to the *pusillus* group and can be distinguished by its notched, broadly-based triangular connecting process, which is typically blunt, but may occasionally appear sharply pointed (Peters 1871, Csorba et al. 2003). The posterior margin of the connecting process is directly attached to the face of the posterior noseleaf. The lancet displays concave lateral margins that become nearly parallel towards the tip (Bates et al. 2004), while the sella is parallel-sided and covered with fine hairs (Kingston et al. 2009). The skull is relatively broad with a short rostrum and the upper canine is slender, often narrowing beyond the cingulum. The forearm length ranges from 45 to 53 mm, making *R. acuminatus* noticeably larger than other members of the *pusillus* group, which also share the triangular-shaped connecting process (Kingston et al. 2009). Subspecies variation is minimal and is primarily associated with differences in the shape of the sella and the width of the horseshoe (Francis 2019). The morphologically similar *R. affinis* can be easily distinguished by its rounded connecting process (Francis 2019).

This species emits an FM/CF/FM echolocation call, a typical acoustic characteristic of the genus *Rhinolophus*. However, the peak constant frequency (CF_Peak_) varies considerably amongst populations across different regions. In Laos, CF_Peak_ values range from 86–90 kHz in males and 93–95 kHz in females. In Vietnam, a peak frequency of around 90 kHz has been recorded, while in Thailand, it is approximately at 80 kHz (Csorba et al. 2019). In Malaysian Borneo, individuals from Sabah exhibit CF_Peak_ values between 88–90 kHz (Csorba et al. 2019). In contrast, more recent recordings from Gunung Mulu National Park, Sarawak (GMNP), in northwest Borneo, by McArthur and Anwarali Khan (2021) and ChiroVox (Görföl et al. 2022), indicate slightly lower frequencies, ranging from 82–87 kHz.

This pattern suggests a gradual decline in CF_Peak_ from northern mainland Asia to the southern parts of the species’ range. Additionally, within Malaysian Borneo, a similar trend is observed, with higher frequencies recorded in the north (Sabah) compared to the northwest (Sarawak). These regional differences in echolocation frequency may reflect ecological variation, population structure or even morphological differences, such as body size, which is often inversely correlated with CF_Peak_ in *Rhinolophus* species. A single male individual from Melilas, Brunei is represented in the GBIF database, with associated COI sequence data published in BOLD Systems (The International Barcode of Life Consortium 2024). However, no echolocation data are currently available for populations in Brunei, leaving a gap in our understanding of acoustic variation in this part of Borneo.

**Figure 2.**
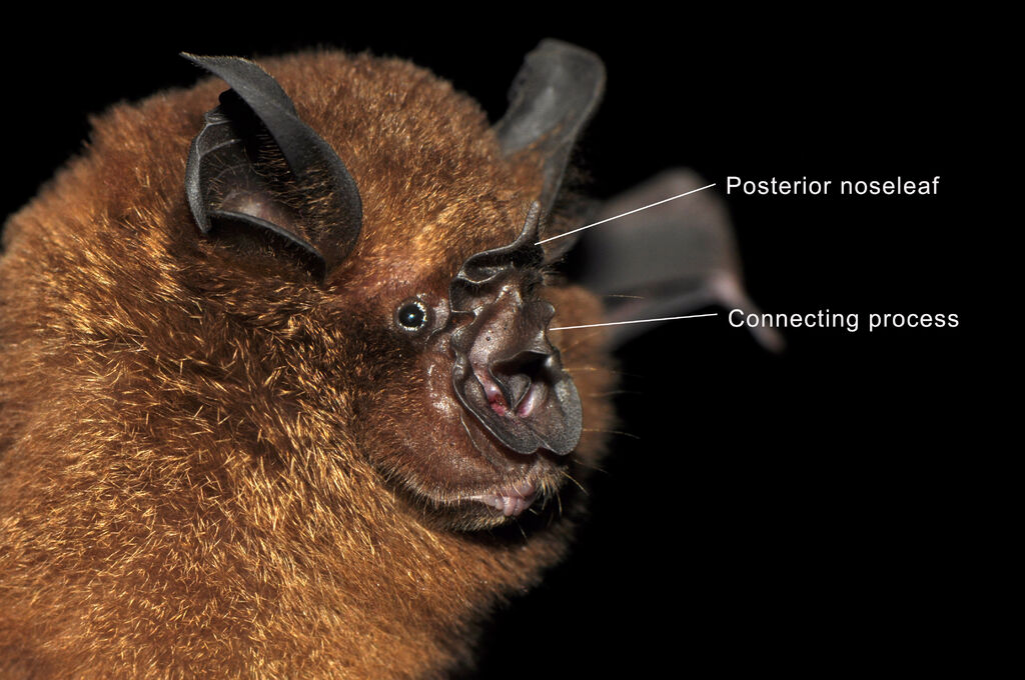
Oblique view of the noseleaf structure of *Rhinolophus acuminatus* from Royal Belum State Park, Malaysia. Photographed by Juliana Senawi, 16 June 2017.

**Figure 3.**
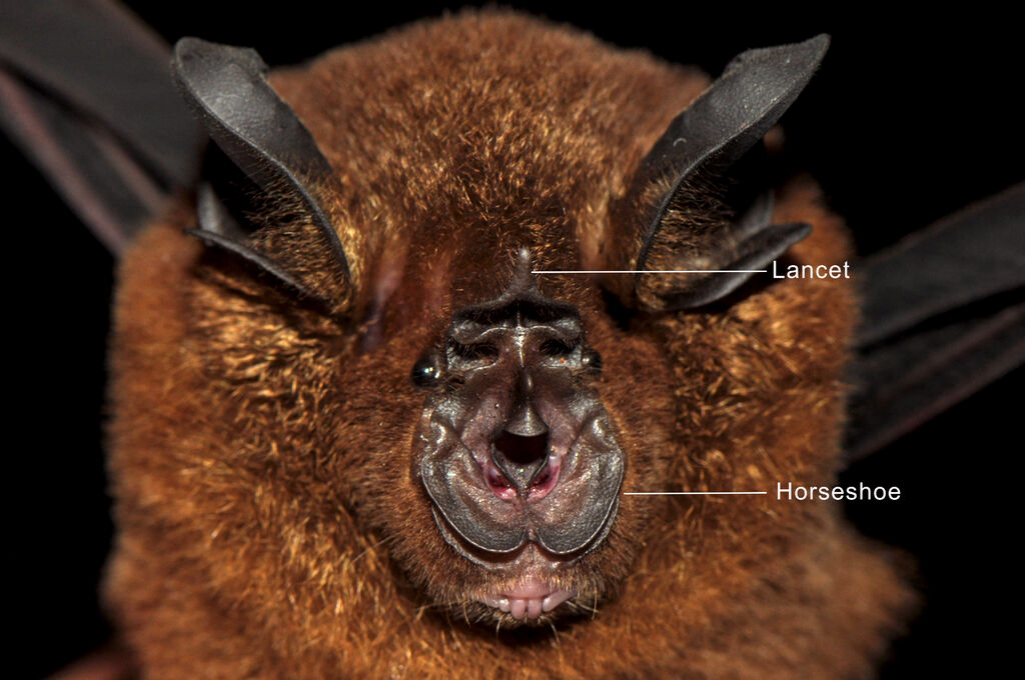
Anterior view of the noseleaf structure of *Rhinolophus acuminatus* from Royal Belum State Park, Malaysia. Photographed by Juliana Senawi, 16 June 2017.

**Figure 4.**
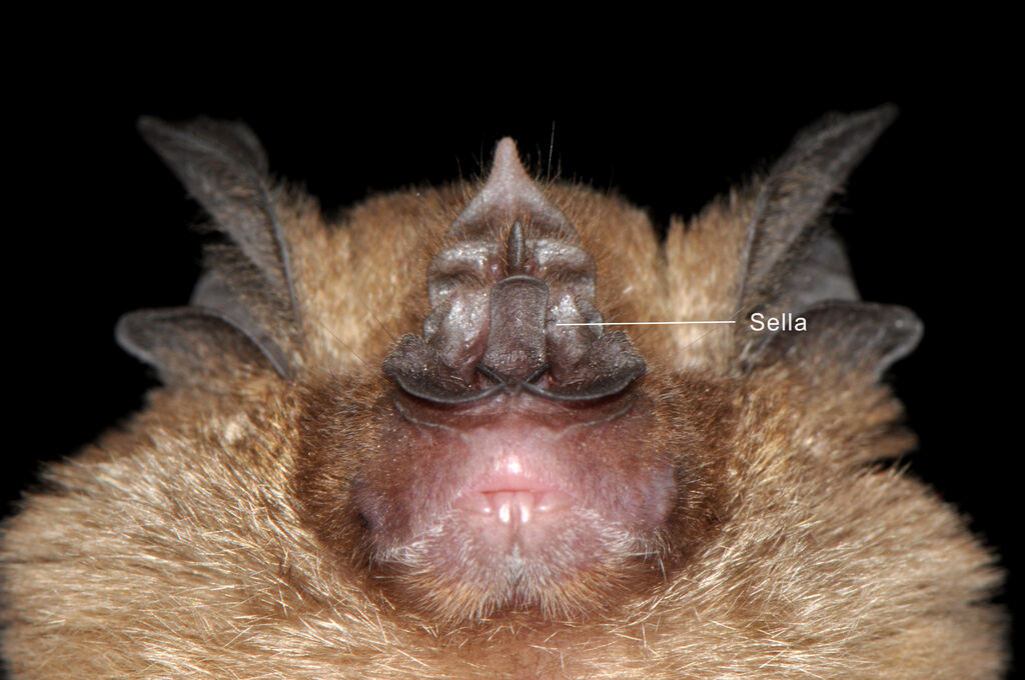
Parallel-sided sella of the noseleaf structure of *Rhinolophus acuminatus* from Royal Belum State Park. Photographed by Juliana Senawi, 16 June 2017.

Figure(s) or Photo(s): Figs 2, 3, 4

Region for assessment: Global

#### Editor & Reviewers

##### Reviewers

Reviewers: Waldien, D.L.

##### Editor

Editor: Zariman, N. F. & Senawi, J.

#### Geographic range

Biogeographic realm: Indomalayan

Countries: Viet NamPhilippinesBrunei DarussalamCambodiaThailandLao People's Democratic RepublicMalaysiaIndonesiaMyanmar

**Figure 5.**
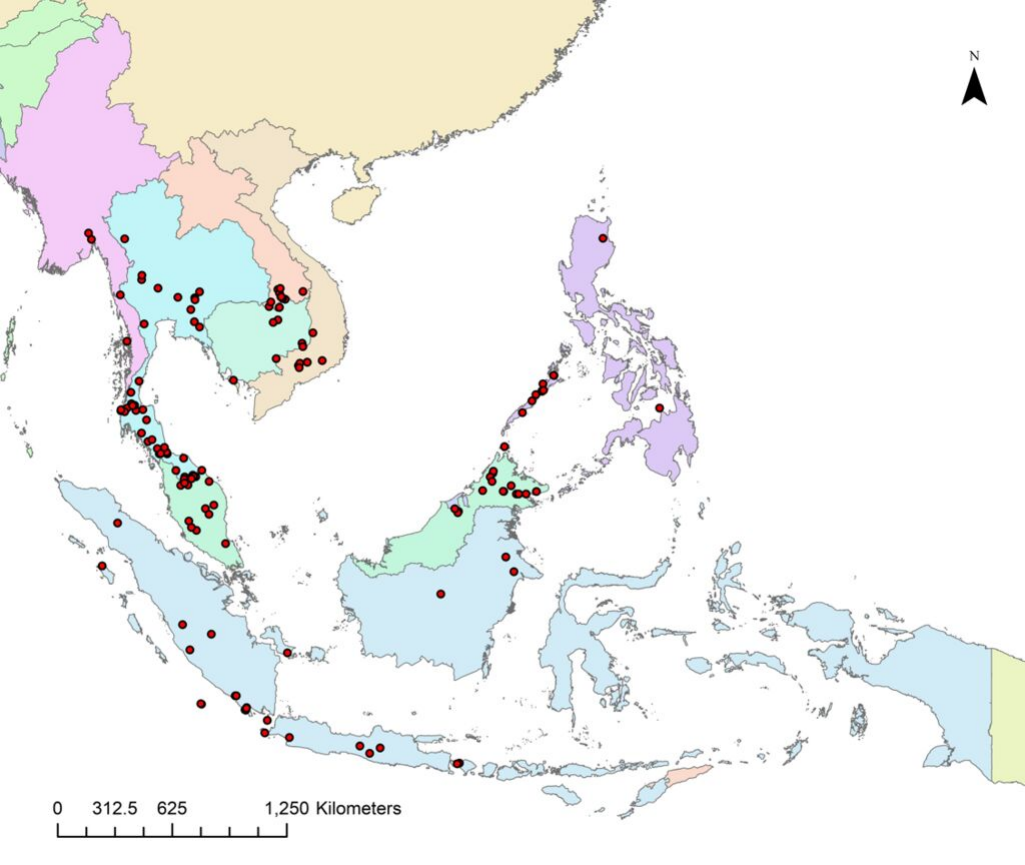
Distribution map of *Rhinolophus acuminatus* using locality data. Symbol: dots – locality points.

Map of records (image): Fig. 5

Map of records (Google Earth): Suppl. material 1

Basis of EOO and AOO: Observed

Basis (narrative): Koopman (1982) recognised five subspecies of *R. acuminatus*, reflecting its wide geographic distribution: *R. a. acuminatus* from Gadok, Java and Krakatau Island; *R. a. sumatranus* from Lower Langkat, North Sumatra and North Borneo; *R. a. audax* from Lombok, Sunda and Bali Island; *R. a. calypso* from Enggano Island, West Sumatra; and *R. a. circe* from Nias Island, West Sumatra (Koopman 1982, Csorba et al. 2003). The species is native to the region and is not known to have been introduced beyond its natural range. It occupies a broad elevational range, from 20 to 3,943 m above sea level and is capable of adapting to a variety of habitats, including lowland dipterocarp forests, upper montane forests and even urban environments. Its estimated Extent of Occurrence (EOO) is approximately 6,957,361.5 km², while its Area of Occupancy (AOO) is 608 km². In the present study, a total of 153 localities were recorded. Based on identified threats, such as habitat loss, 149 discrete locations were delineated. However, *R. acuminatus* is not considered severely fragmented, as most known populations are distributed across the contiguous mainland and larger islands within its range.

Min Elevation/Depth (m): 20

Max Elevation/Depth (m): 3943

Range description: *Rhinolophus acuminatus* is widely distributed across Southeast Asia, with its first formal description originating from Gadok, Java, Indonesia (Csorba et al. 2003). In Peninsular Malaysia, the species has been extensively documented through a range of published studies (Hill 1974, Tamrin et al. 2008, Kingston et al. 2009, Ramli and Hashim 2009, Joann et al. 2011, Abdullah Halim 2014, Lim et al. 2014, Kumaran et al. 2015, Aihara et al. 2016, Trombone 2016, Lim et al. 2019, Nasir 2019, Jayaraj et al. 2020, Munian et al. 2020, Abdullah et al. 2021, Fauzi et al. 2024, Conroy 2025, Lim et al. 2025), as well as from original data collected during the present study in 2005, 2013, 2017 and 2020 (Suppl. material 2). In Malaysian Borneo, the species has likewise been recorded in peer-reviewed publications (Tuen et al. 2002, Ketol et al. 2009, Bansa et al. 2020, McArthur and Anwarali Khan 2020, Senawi et al. 2020, Yoh et al. 2020, McArthur and Anwarali Khan 2021), through data collected during the present study in 2015 (Suppl. material 2), as well as through biodiversity databases (GBIF 2024, iNaturalist contributors, iNaturalist 2024, Observation.org 2024) and institutional records (Harvard University Museum, Morris P J 2024, Pestridge 2024). Beyond Malaysia, the species has been reported from Cambodia (Walston et al. 2001, Matveev 2005), Laos (Robinson 1998, Thomas et al. 2013), Indonesia (Schedvin et al. 1994, Suyanto et al. 1998, Suyanto and Struebig 2007, Lestari et al. 2013, Huang et al. 2014, Syamsi 2015, Grant et al. 2020, Huang et al. 2020, Ševčík et al. 2022, Harvard University Museum, Morris P J 2024, Orrell T, Informatics and Data Science Center - Digital Stewardship 2024, Kalthoff 2025), Vietnam (Borissenko and Kruskop 2003, Thong 2015, Thong et al. 2021, Görföl et al. 2022, European Bioinformatics Institute (EMBL-EBI) 2024), the Philippines (Esselstyn et al. 2004, Cabauatan et al. 2014, Gonzales et al. 2014, Lobite 2017, Grant et al. 2020, Tanalgo 2023, Bentley 2024, Tanalgo and Dela Cruz 2024), Thailand (Sanborn 1952, Harada et al. 1982, Hood et al. 1988, Qumsiyeh et al. 1988, Phommexay et al. 2011, Soisook et al. 2016, Aroon et al. 2016, Samoh et al. 2021, Wacharapluesadee et al. 2021, Boonchuay and Bumrungsri 2022, GBIF 2024, iNaturalist contributors, iNaturalist 2024, Observation.org 2024, Saelao and Soisook 2024, Wildife Yearbook 2024, Conroy 2025, Kanphan et al. n.d.), Myanmar (Bates et al. 2004, GBIF 2024) and Brunei (iNaturalist contributors, iNaturalist 2024).

#### Extent of occurrence

EOO (km2): 6,957,361.5

Trend: Stable

**Figure 6.**
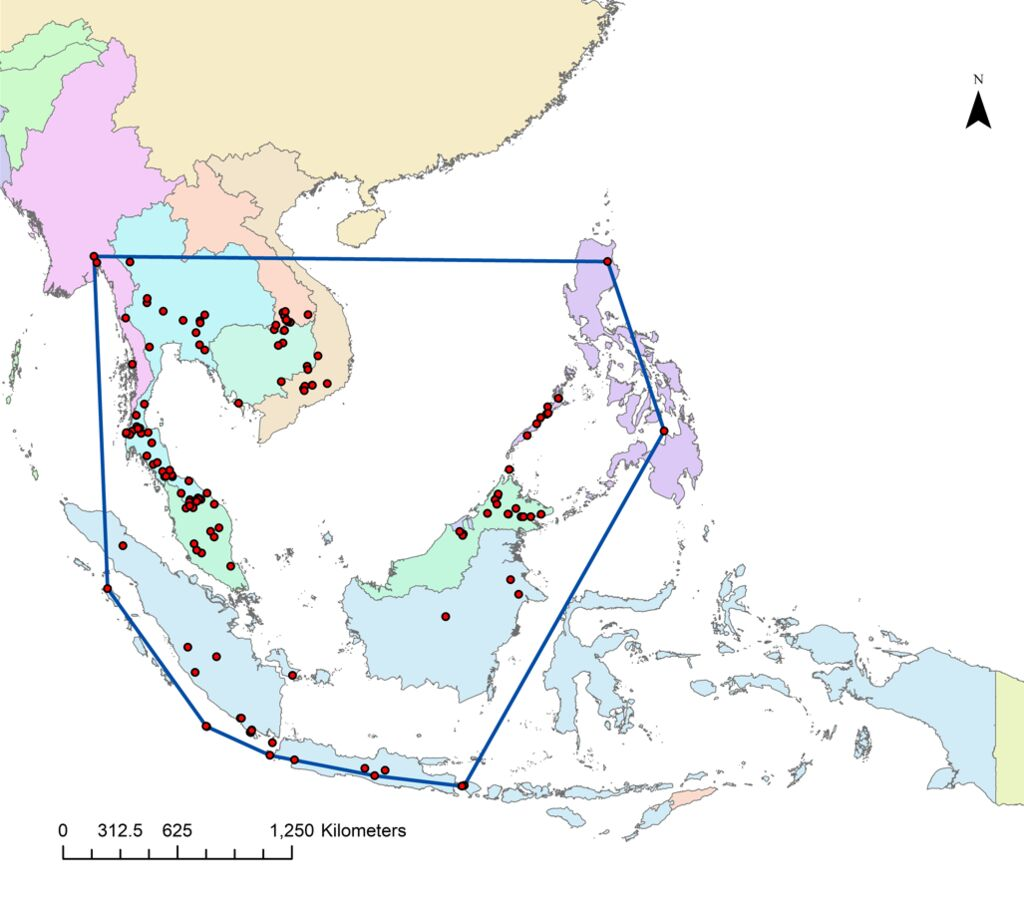
The estimated Extent of Occurrence (EOO) for *Rhinolophus acuminatus* is approximately 6,957,361.5 km².

Justification for trend: Based on the estimated Extent of Occurrence (EOO), *Rhinolophus acuminatus* is found across Southeast Asian countries, with the exception of Singapore and Timor-Leste. Currently, there is no evidence of any reduction in the species' EOO (Fig. 6)

Causes ceased?: No

Causes understood?: Yes

Causes reversible?: No

Extreme fluctuations?: No

#### Area of occupancy

Trend: Decline (observed)

Justification for trend: Approximately 24% of its Area of Occupancy (AOO) occurs within modified habitats, such as plantations and fragmented forests. These habitat types pose substantial threats to the species’ foraging areas and overall habitat quality.

Causes ceased?: No

Causes understood?: Yes

Causes reversible?: No

Extreme fluctuations?: No

AOO (km2): 608

#### Locations

Number of locations: 149 points

Justification for number of locations:

The number of locations was determined, based on the most plausible threat to the species, which is habitat loss. Although 153 localities have been recorded (Suppl. material 2), the number of locations is considered to be 149.

In Gunung Mulu National Park, three localities are recorded, but two of them (Rhac031 and Rhac032) are likely to be affected by a single threatening event, as they are situated within the same large continuous forest. Therefore, the number of locations was revised from 153 to 151. Gunung Mulu National Park is recognised as one of the best-protected and best-managed protected areas in Southeast Asia and is assessed as “Low Concern” under Criteria (vii), (viii), (ix) and (x) of the IUCN Conservation Outlook Assessment.

Similarly, the two locality points in Cat Tien National Park (Rhac089 and Rhac093) are considered a single location, as they are likely to be affected by a single threatening event, reducing the total to 150. Additionally, two of the six locality points in Bukit Barisan Selatan National Park (Rhac060 and Rhac081) are treated as a single location for the same reason. Bukit Barisan Selatan National Park holds the highest level of protection under Indonesian law, although it is listed as “In Danger” in the IUCN Conservation Outlook Assessment. Given the spatial separation of these sites and the generally high level of protection in place, it is considered unlikely that a single threatening event would affect all five sites. In conclusion, the number of locations is assessed as 149.

Trend: Stable

Extreme fluctuations?: No

#### Population

Trend: Unknown

Justification for trend: Currently, there is no detailed population data available for *Rhinolophus acuminatus* and its overall population trend remains unknown. However, some localised observations provide insight into its social behaviour. In southern Vietnam, the species has been recorded roosting in colonies of up to approximately 500 individuals (Thong et al. 2019), while in Myanmar, colony sizes of around 100 individuals have been reported (Bates et al. 2004).

Causes ceased?: Unknown

Causes understood?: Unknown

Causes reversible?: Unknown

Extreme fluctuations?: Unknown

#### Subpopulations

Number of subpopulations: 149

Trend: Unknown

Justification for trend: As no population studies have been conducted on this species, information on its subpopulations is currently lacking. However, the number of subpopulations is inferred from the number of known locations, following the IUCN Assessment Template (Version 2023).

Extreme fluctuations?: No

Severe fragmentation?: No

Justification for fragmentation: This species is distributed across various nature parks and wildlife reserves throughout its entire range. Most subpopulations are found on the mainland of Southeast Asia, with several additional occurrences on surrounding small islands. Given this distribution, there is no indication of population fragmentation.

#### Habitat

System: Terrestrial

Habitat specialist: No

Habitat (narrative): *Rhinolophus acuminatus* has been documented in a variety of habitats, including tropical dry forests, tropical moist lowland forests, tropical moist montane forests, caves and, to a lesser extent, urban areas (Thong 2015, Thong et al. 2019, Munian et al. 2020, McArthur and Anwarali Khan 2021). In southern Vietnam, this species has been observed roosting in large colonies of up to 500 individuals in caves, while foraging in surrounding forests for small insects (Thong et al. 2019). In contrast, in Peninsular Malaysia, *R. acuminatus* has been found roosting singly or in pairs under palm trees and in residential buildings (Kingston et al. 2009). A study in Thailand investigating the impact of plantations on insectivorous bats reported that *R. acuminatus* was captured exclusively in forested areas, unlike other *Rhinolophids*, such as *R. affinis*, which were found in both forest and plantation habitats (Phommexay et al. 2011).

Trend in extent, area or quality?: Unknown

##### Habitat

Habitat importance: Major Importance

Habitats: 1.5. Forest - Subtropical/Tropical Dry1.6. Forest - Subtropical/Tropical Moist Lowland1.9. Forest - Subtropical/Tropical Moist Montane7.1. Caves and Subterranean Habitats (non-aquatic) - Caves

#### Ecology

Size: Head and body length: 54.5 ± 4.5 mm, Tail 26 ± 4.0 mm, Forearm 48.0 ± 3.0 mm, Tibia 20.5 ± 2.5 mm, Ear length 21.5 ± 3.5 mm, Weight 13.0 ± 3.0 g.

Generation length (yr): 10

Dependency of single sp?: No

Ecology and traits (narrative):

*Rhinolophus acuminatus* is a viviparous mammal, giving birth to live young. Breeding observations in Peninsular Malaysia have recorded pregnant individuals in February and April (Kingston et al. 2009). However, specific life history data, such as lifespan and age at sexual maturity, are lacking for this species. Generation length is, therefore, estimated at 10 years, based on an assumed lifespan of 20 years and an age of sexual maturity at 2 years, consistent with other species in the genus *Rhinolophus* (Jones et al. 2009).

Insectivorous bats, including *R. acuminatus*, are capable of consuming up to 125% of their body weight in insects per night, with pregnant and lactating females exhibiting the highest feeding rates compared to males and non-lactating females (Kasso and Balakrishnan 2013). Through its diet of small insects, *R. acuminatus* plays a vital role in regulating insect populations, including agricultural pests and vectors.

#### Threats

Justification for threats:

Currently, no major threats have been identified for this species, either directly or indirectly. Under the IUCN threat classification framework, major threats are defined as those affecting the species most important habitats, typically roost sites. In this assessment, most known localities (97%) represent foraging sites. Only one confirmed cave roost site has been recorded, located within a protected area (Cat Tien National Park), which is included in the Tentative List under the IUCN Conservation Outlook Assessment. However, localised threats such as cave disturbance, including mining and quarrying activities, have been reported to negatively affect populations (Thong et al. 2019). In contrast, in Myanmar, colonies have been observed roosting in man-made structures such as a cellar beneath a monastery, the roof of a prison and the basement of a townhouse, where localised disturbances, such as building modifications and upgrades, pose potential risks to roosting colonies.

Potential major threats also include any disturbances to their foraging and roosting sites. For example, Phommexay et al. (2011) reported that *R. acuminatus* was absent from plantation areas near forests, suggesting that such land use may act as barriers to the species’ foraging grounds, thereby impacting its feeding behaviour. Additionally, studies on the effects of road lighting indicate that bats tend to avoid illuminated areas, effectively reducing their available foraging habitat (Pauwels et al. 2021).

##### Threats

Threat type: Ongoing

Threats: 1.2. Residential & commercial development - Commercial & industrial areas2.1.3. Agriculture & aquaculture - Annual & perennial non-timber crops - Agro-industry farming3.2. Energy production & mining - Mining & quarrying4.1. Transportation & service corridors - Roads & railroads6.1. Human intrusions & disturbance - Recreational activities9.6.1. Pollution - Excess energy - Light pollution5.3.4. Biological resource use - Logging & wood harvesting - Unintentional effects (large scale)

## Use and Trade

**General Use and Trade Information**:

Although there is no evidence of *R. acuminatus* being used or traded in the region, the practice of using bats as souvenirs is relatively widespread, suggesting a high potential for this species to be exploited in a similar manner.

Use and Trade

## Conservation

**Conservation type**: In Place

**Conservation actions**:

1.1 Land/Water protection: site/area protection

**Conservation actions needed**:

4.3 Education & Awareness: Awareness & Communications

**Justification for conservation actions**:

Of the 149 confirmed localities for this species, more than half are located within protected areas, such as forest reserves, wildlife reserves and nature reserves. Monitoring and safeguarding these habitats are vital for the species' conservation in Southeast Asia. However, communication, education and public awareness about bats, especially in this particular species, remain limited amongst government agencies and the general public. Promoting understanding of the ecological roles play by bats, including ecosystem services, pest and vector regulation, can enhance public appreciation of their importance. Public education on threats, such as habitat loss is also key to building support for conservation efforts. Strengthening collaboration amongst government agencies, conservation organisations and local communities is vital to ensure the long-term stability of the species' population in the region.

**Conservation sites identified**: Yes

**Occur in at least one Protected Area**: Yes

Conservation

## Research Actions

**Research actions needed**:

1.1 Research: Taxonomy

1.2 Research: Population size, distribution & trends

3.1 Monitoring: Population trends

**Justification for research actions needed**:

There are 153 known localities of *R. acuminatus*, with 149 number of locations. The majority of these sites are located within protected areas, such as forest reserves and state parks, which offer a degree of protection for many populations. However, to ensure the long-term survival of this species, additional conservation efforts are needed, including improved land-management practices and increased public awareness.

In Malaysia, the designation of forest reserves is not permanent, as state governments can reclassify these areas for ‘higher economic use’. Several forest reserves have already been converted for large-scale developments, including residential projects, highways and dams. Raising awareness amongst the public and policy-makers about the vital ecosystem services provided by insectivorous species like *R. acuminatus* is essential for protecting both their habitats and population stability.

Although *R. acuminatus* is distributed throughout Southeast Asia, there is a significant lack of data on its population size. Collaborative efforts from bat researchers across the region are crucial for generating this information, particularly to assess population trends and guide future conservation strategies.

Research Actions

## IUCN Red List Assessment

The Extent of Occurrence (EOO) for *Rhinolophus acuminatus* is estimated at 6,957,361.5 km² and its Area of Occupancy (AOO) is 608 km². According to IUCN Red List Criterion B (Geographic Range), the thresholds for a threatened category are an EOO of less than 20,000 km² or an AOO of less than 2,000 km². As *R. acuminatus* exceeds these thresholds, it does not qualify for listing under any threatened category, based on this criterion.

*Rhinolophus acuminatus* is, therefore, assessed as Least Concern (LC), supported by its wide distribution and occurrence in numerous protected areas throughout its range. Currently, there are no major threats or observed population declines that would justify a higher risk category under the IUCN Red List Categories and Criteria.

IUCN Red List Assessment

## Conclusion

In conclusion, this study underscores the critical role of comprehensive locality data in accurately assessing the conservation status of *Rhinolophus acuminatus*. This includes information on the Extent of Occurrence (EOO), Area of Occupancy (AOO), number of occupied locations and observations of declines or fluctuations in these parameters as well as in the number of mature individuals. By integrating field records with an exhaustive literature review, we have expanded the known distribution of the species and quantified key range metrics, laying the groundwork for more robust evaluations of population stability and threat exposure. These newly-identified sites provide essential baselines for detecting future trends. Moving forward, targeted studies on roosting ecology, population size estimates and long-term monitoring are imperative to fill the remaining knowledge gaps. Ultimately, this enhanced dataset not only refines the IUCN Red List assessment for *R. acuminatus*, but also helps to prioritise and guide the most effective research and conservation actions.

Conclusion

## Supplementary Material

A4592B68-FCA3-5662-B30F-1D5603C54871 10.3897/BDJ.13.e162374.suppl1 Supplementary material 1 Locality data of *Rhinolophus acuminatus* Data type map File: oo_1457832.kml https://binary.pensoft.net/file/1457832 Nazifah Fitriyah Zariman, Juliana Senawi

4348D869-067B-5F5F-9435-C54E953E3872 10.3897/BDJ.13.e162374.suppl2 Supplementary material 2 List of localities and data sources of Rhinolophus acuminatus Data type occurrences File: oo_1379189.pdf https://binary.pensoft.net/file/1379189 Nazifah Fitriyah Zariman, Juliana Senawi
