# Supplementary material for: Species conservation profile and revision of Rhinolophus acuminatus (Chiroptera, Rhinolophidae) from Southeast Asia
Source: Biodivers Data J. 2025 Nov 28;13:e162374. doi: 10.3897/BDJ.13.e162374 (PMC12680947; doi:10.3897/BDJ.13.e162374)
Supplement: Supplementary material 2 — List of localities and data sources of Rhinolophus acuminatus [file bdj-13-e162374-s002.pdf]

Supplementary: List of localities and data sources of *Rhinolophus acuminatus*

Country	No	Code	Longitude	Latitude	Source
Malaysia	1	Rhac001	6.119962	102.246294	iNaturalist, 2024
	2	Rhac002	5.98333	116.533333	Jayaraj V K, Aminuddin-Baqi M H F, Iqbal M B N H, Nur-Nabilah B A R, Azhar I, Ean W C, Anwarali Khan F A (2020). Bats of Northern Peninsular Malaysia. Version 1.4. Southeast Asian Bat Conservation Research Unit. Occurrence dataset https://doi.org/10.15468/4dtajy accessed via GBIF.org on 2025-06-09. https://www.gbif.org/occurrence/2640445535
	3	Rhac003	6.0801	116.5556	Lim L S, Huang J C, Anuar S, Struebig M, Rossiter S (2025). Forest bats in Peninsular Malaysia_2007-2009. Version 1.6. Department of Life Science, National Taiwan Normal University. Occurrence dataset https://doi.org/10.15468/vatum8 accessed via GBIF.org on 2025-06-09. https://www.gbif.org/occurrence/4999844650
	4	Rhac004	7.283333	117.099999	Trombone T (2016). AMNH Mammal Collections. American Museum of Natural History. Occurrence dataset https://doi.org/10.15468/wu3poe accessed via GBIF.org on 2025-06-09. https://www.gbif.org/occurrence/859445971
	5	Rhac005	5.3677	117.4306	Conroy C (2025). MVZ Mammal Collection (Arctos). Version 35.95. Museum of Vertebrate Zoology. Occurrence dataset https://doi.org/10.15468/uwudf9 accessed via GBIF.org on 2025-06-09. https://www.gbif.org/occurrence/5147325572
	6	Rhac006	4.96184	117.689216	Observation.org (2025)
	7	Rhac007	4.962963	117.802642	iNaturalist, 2024
	8	Rhac008	5.083	118.667	McArthur E, Anwarali Khan F A (2020). Insectivorous Bats of Gunung Mulu National Park, Malaysia. Version 1.6. Southeast Asian Bat Conservation Research Unit. Occurrence dataset https://doi.org/10.15468/2tbfez accessed via GBIF.org on 2025-06-09. https://www.gbif.org/occurrence/2640277324
	9	Rhac009	4.10637	114.84511	Grant S, Webbink K, Jones J, Ferguson A (2020). Field Museum of Natural History (Zoology) Mammal Collection. Version 9.18. Field Museum. Occurrence dataset https://doi.org/10.15468/n4zgxw accessed via GBIF.org on 2025-06-09. https://www.gbif.org/occurrence/665923916
	10	Rhac010	5.383333	101.216667	Harvard University M, Morris P J (2025)
	11	Rhac011	6.318567	100.483789	Lim, L.S., Struebig, M.J., Zalipah, M.N., Mohd-Adnan, A., Senawi, J., Zubaid, A., Mohd Sah, S.A. and Rossiter, S.J., 2019. Bats from the understorey of lowland tropical rainforests across Peninsular Malaysia. Journal of Bat Research and Conservation, 12(1), pp.68-82.
	12	Rhac012	5.669683	102.168325	
	13	Rhac013	4.412189	102.682014	
	14	Rhac014	4.137861	103.359165	
	15	Rhac015	6.115262	100.9786	Present study: Fieldwork (2020)
	16	Rhac016	3.629942	101.614239	Munian, K., Azman, S. M., Ruzman, N. A., Fauzi, N. F. M. & Zakaria, A. N. 2020. Diversity and composition of volant and non-volant small mammals in northern Selangor State Park and adjacent forest of Peninsular Malaysia. Biodiversity Data Journal. 8:e50304.
	17	Rhac017	2.529225	103.414567	Aihara, Y., Hosaka, T., Yasuda, M., Hashim, M. and Numata, S., 2016. Mammalian wildlife tourism in South-east Asian tropical rainforests: the case of Endau Rompin National Park, Malaysia. Journal of Tropical Forest Science, pp.167-181.

	18	Rhac018	4.239274	102.429287	Abdullah, N.I., Khodri, N.F., Arifin, N.A.T., Rahim, M.R.A., Razali, S.H.A., Darbis, N.D.A. and Nor, S.M., 2021. Small Mammals of Tekai Tembeling Forest Reserve (TTFR), Jerantut, Pahang, Peninsular Malaysia. Journal of Sustainable Natural Resources, 2(2), pp.12-21.
	19	Rhac019	4.414087	102.834091	Lim, L.-S., Mohd-Adnan, A., Zubaid, A., Struebig, M. J. & Rossiter, S. J. 2014. Diversity of Malaysian insectivorous bat assemblages revisited. Journal of Tropical Ecology. 30(02).
	20	Rhac020	3.968283	102.599233	Abdullah Halim, Muhammad Rasul (2014) A comparative study of small mammal diversity of oil palm plantation and nearby forest / Muhammad Rasul bin Abdullah Halim. Masters thesis, University of Malaya.
	21	Rhac021	3.720359	102.276751	Kingston et al. 2009. bats of krau wildlife reserve
	22	Rhac022	3.710172	102.287073	Present study: Fieldwork (2013)
	23	Rhac023	5.715547	101.744094	Kumaran, J.V., Anwarali Khan F A., Azhar, I., WeeChen, E., Ali, M.R.M., Ahmad, A. and Yusoff, A.M., 2015. Diversity and conservation status of small mammals in Kelantan, Malaysia.
	24	Rhac024	5.405493	101.566313	CL, J., FLETCHER, C., SALIM, H.M., RAHMAN, A., HARRISON, R.D. and POTTS, M.D., 2011. Insectivorous bat assemblage in the hill dipterocarp forest of Temengor Forest Reserve, Peninsular Malaysia. Malayan Nature Journal, 63(3), pp.569-576.
	25	Rhac025	5.714443	101.7428267	GBIF, 2024
	26	Rhac026	5.792159	101.406782	Tamrin, N.A.M., Rahman, M.R.A., Daud, M.H.R.M., Hassan, N. and Abdullah, M.T., 2008, December. A survey on small mammals in the Royal Belum Park, Perak, Malaysia. In Proceeding of National Biodiversity Seminar (pp. 7-14).
	27	Rhac027	5.704535	101.324205	Hill, J. E., 1974. New Records of of bats from Southeastern Asia with taxonomic notes. Bull Br Mus Nat Hist Zool. 27:127-138
	28	Rhac028	5.686374	101.377264	Present study: Fieldwork (2005)
	29	Rhac029	5.809005	101.423703	Present study: Fieldwork (2017)
	30	Rhac030	5.579173	102.606729	Fauzi, N.A., Munian, K., Mahyudin, N.A.A., Rosdy, F.N.M. and Ahmad, M.B., 2024. Small mammal assemblage in Gunung Tebu Forest Reserve, Terengganu: An environmentally sensitive area in East Coast Peninsular Malaysia. In BIO Web of Conferences (Vol. 131, p. 01005). EDP Sciences.
	31	Rhac031	4.042139	114.8149	McArthur, E. and Anwarali Khan F A., 2021. Towards a regional call library: Classifying calls of a species-rich bat assemblage in a Bornean karst rainforest. *Journal of Bat Research & Conservafion Volume*, 14(1):95-117
	32	Rhac032	4.042245	114.814446	Present study: Fieldwork (2015)
	33	Rhac033	3.179886	101.985822	Ramli, R. & Hashim, R. 2009. Diversity of small mammals inhabiting disturbed forest: A case study on Kenaboi Forest Reserve, Jelebu, Negeri Sembilan, Malaysia. Malaysian Journal of Science. 28(4):481-490
	34	Rhac034	3.31985	101.7418667	Nasir, N.M., 2019. Diversity of Bats in Three Selected Forest Types in Selangor and Kuala Lumpur (Master's thesis, University of Malaya (Malaysia)).

	35	Rhac035	5.79812	116.405215	Yoh, N., Azhar, I., Fitzgerald, K.V., Yu, R., Smith-Butler, T., Mahyudin, A. and Kingston, T., 2020. Bat ensembles differ in response to use zones in a tropical biosphere reserve. Diversity, 12(2), p.60.
	36	Rhac036	5.575381	116.490743	Tuen, A.A., Lakim, M.B. and Hall, L.S., 2002. Preliminary survey of bats of the Crocker Range National Park Sabah, Malaysia. ASEAN Review of Biodiversity and Environmental Conservation (ARBEC) July-September.
	37	Rhac037	4.963099	118.164579	Ketol, B., Anwarali, F.A., Marni, W., Sait, I., Lakim, M., Yambun, P.I., Salleh, M.A., Rahman, M.A. and Abdullah, M.T., 2009. Checklist of mammals from Gunung Silam, Sabah, Malaysia. Journal of Tropical Biology & Conservation (JTBC), 5.
	38	Rhac038	5.091012	117.065293	Bansa, L.A., Rosli, Q.S., Daud, U.S., Amat, A., Morni, M.A., Dee, J.W., Jinggong, E.R., Rajasegaran, P., Senawi, J., Kumaran, J.V. and Azhar, I., 2020. Survey on the Small Mammals in Sg. Kangkawat Research Station Imbak Canyon Conservation Areas. Journal of Tropical Biology & Conservation (JTBC), 17, pp.149-163.
	39	Rhac039	5.093669	117.039753	Senawi, J., Mahyudin, A., Daud, U. S., Amat, A., Lagundi, S., Gondilang, E., ... & Azhar, I. (2020). Bat diversity in Imbak Canyon Conservation Area: Note on their echolocation calls and ectoparasites. Journal of Tropical Biology & Conservation (JTBC), 17, 217-232.
	40	Rhac040	5.130782	116.031698	Lok, Y.C., Siau, V.G., Kanapiah, N.A.A.M., Lai, T.C., Haslan, N.N.H.H., Nukili, N.N., Daud, U.S., Amat, A., Gompoyo, J., Tuh, Y.Y.F. and Hasan, N.H., 2021. Bat species diversity trend along an elevation gradient: A study in Crocker Range Park, Sabah, Borneo. Biodiversity Data Journal, 9.
Cambodia	41	Rhac041	10.54451	103.79838	GBIF, 2024
	42	Rhac042	13.502313	105.980304	Matveev, V.A., 2005. Checklist of Cambodian bats (Chiroptera), with new records and remarks on taxonomy. Russian Journal of Theriology. Русский териологический журнал, 4(1):43-62.
	43	Rhac043	13.381538	105.749947	
	44	Rhac044	12.358953	107.168633	Walston, J., Davidson, P., Soriyun, M. and NY) Wildlife Conservation Society (New York Cambodia Program, 2001. A wildlife survey of southern Mondulkiri province, Cambodia. Wildlife Conservation Society Cambodia Program.
Lao PDR	45	Rhac045	14.158	105.55	GBIF, 2024
	46	Rhac046	14.383	105.633	GBIF, 2024
	47	Rhac047	15	105.983	GBIF, 2024
	48	Rhac048	14.822	106.06	GBIF, 2024
	49	Rhac049	15.05	106.1	GBIF, 2024
	50	Rhac050	14.5152778	106.3430556	Robinson, M. F. 1998. Chiroptera Survey: Xe Pian National Biodiversity Conservation Area, Lao PDR. Natural history bulletin of the siam society. 46: 155-170.
	51	Rhac051	14.5625	106.2125	
	52	Rhac052	14.6305556	106.1277778	
	53	Rhac053	14.6402778	106.1513889	
	54	Rhac054	14.1111111	106.0388889	
	55	Rhac055	14.1208333	106.0611111	
	56	Rhac056	14.891676	107.211069	Thomas, N. M., Duckworth, J. W., Douangboubpha, B., Williams, M., & Francis, C. M. (2013). A Checklist of Bats

Supplementary: List of localities and data sources of *Rhinolophus acuminatus*

					(Mammalia: Chiroptera) from Lao PDR. Acta Chiropterologica, 15(1), 193–260. doi:10.3161/150811013x667993
Indonesia	57	Rhac057	-5.354104	102.230863	iNaturalist, 2024
	58	Rhac058	-5.334557	102.200261	Huang J C C, Kingston T, Rustiati E L, Nusalawo M (2020). Bats of Bukit Barisan Selatan Landscape, Sumatra, Indonesia. Version 1.6. Southeast Asian Bat Conservation Research Unit. Occurrence dataset https://doi.org/10.15468/emgg92 accessed via GBIF.org on 2025-06-10. https://www.gbif.org/occurrence/2640458739
	59	Rhac059	-4.942176	103.88358	
	60	Rhac060	-4.94	103.94	Harvard University M, Morris P J (2025). Museum of Comparative Zoology, Harvard University. Version 162.475. Museum of Comparative Zoology, Harvard University. Occurrence dataset https://doi.org/10.15468/p5rupv accessed via GBIF.org on 2025-06-10. https://www.gbif.org/occurrence/476867900
	61	Rhac061	-8.248643	114.886771	Grant S, Webbink K, Jones J, Ferguson A (2020). Field Museum of Natural History (Zoology) Mammal Collection. Version 9.18. Field Museum. Occurrence dataset https://doi.org/10.15468/n4zgxw accessed via GBIF.org on 2025-06-10. https://www.gbif.org/occurrence/665803474
	62	Rhac062	1.43157	97.3535	Orrell T, Informatics and Data Science Center - Digital Stewardship (2025). NMNH Extant Specimen Records (USNM, US). Version 1.94. National Museum of Natural History, Smithsonian Institution. Occurrence dataset https://doi.org/10.15468/hnhrg3 accessed via GBIF.org on 2025-06-10. https://www.gbif.org/occurrence/1317600431
	63	Rhac063	-5.65	104.4	Kalthoff D C (2025). NRM-Mammals. Swedish Museum of Natural History. Occurrence dataset https://doi.org/10.15468/sb6fcq accessed via GBIF.org on 2025-06-10. https://www.gbif.org/occurrence/5004102397
	64	Rhac064	-5.519722	104.45	GBIF, 2024
	65	Rhac065	-2.833501	106.451515	GBIF, 2024
	66	Rhac066	-6.98523	106.54754	GBIF, 2024
	67	Rhac067	-7.404518	110.004444	GBIF, 2024
	68	Rhac068	-7.766334	110.484367	GBIF, 2024
	69	Rhac069	-7.5	111	GBIF, 2024
	70	Rhac070	-8.276928	114.779093	Suyanto, A., Yoneda, M., Maryanto, I. and Maharadatunkamsi & Sugardjito, J., 1998. Checklist of the mammals of Indonesia. JICA Joint Project for Biodiversity Conservation in Indonesia. LIPI, Bogor.
	71	Rhac071	-6.76215	105.338421	
	72	Rhac072	-2.689914	101.662335	
	73	Rhac073	-1.450921	101.3028	Syamsi, F., 2015. DIVERSITY OF MICROCHIROPTERAN BATS IN FOREST FRAGMENTS AND RIPARIAN ZONES IN AN OIL PALM PLANTATION ESTATE. JURNAL DIMENSI, 4(1).
	74	Rhac074	3.533485	98.116128	Ševčík, M., Kalúz, S. and Šrámek, P., 2022. Bat-infesting chiggers (Trombiculidae) in Indonesia: current review, distribution, and hosts with three new records and their morphometric data. Acta Parasitologica, 67(2), pp.892-903.
	75	Rhac075	-1.916563	102.713181	Neil Jun Sala Lobite. 2017. Impacts of Lowland Rainforest Transformationto Rubber and Oil Palm on Multiple Dimensions

					of Chiropteran Diversity. [Master's thesis: University of Philippines Los Banos]
	76	Rhac076	0.053285	113.976254	Suyanto, A., Yoneda, M., Maryanto, I. and Maharadatunkamsi & Sugardjito, J., 1998. Checklist of the mammals of Indonesia. JICA Joint Project for Biodiversity Conservation in Indonesia. LIPI, Bogor.
	77	Rhac077	1.15023	117.567127	Suyanto, A., & Struebig, M. J. (2007). Bats of the Sangkulirang limestone karst formations, East Kalimantan—a priority region for Bornean bat conservation. Acta Chiropterologica, 9(1), 67-95.
	78	Rhac078	1.866944	117.166944	Lestari, N.S., Suryanto, A.S., Atmoko, T., Kusrini, M.D., Pujiansyah, S.R. and Rojikin, A., 2013, July. THE BIODIVERSITY POTENTIAL OF LABANAN RESEARCH FOREST1. In International Conference on Forest and Biodiversity.
	79	Rhac079	-6.149383	105.46251	Schedvin, N. K., Cook, S. P., & Thornton, I. W. B. (1994). The Diversity of Bats on the Krakatau Islands in the Early 1990s. Biodiversity Letters, 2(3), 87. doi:10.2307/2999762
	80	Rhac080	-4.943854	103.8881	Huang, J.C.C., Jazdzyk, E.L., Nusalawo, M., Maryanto, I., Wiantoro, S. and Kingston, T., 2014. A recent bat survey reveals Bukit Barisan Selatan Landscape as a chiropteran diversity hotspot in Sumatra. Acta Chiropterologica, 16(2), pp.413-449.
	81	Rhac081	-4.94	103.939722	
	82	Rhac082	-5.629722	104.37	
	83	Rhac083	-5.650384	104.400075	
	84	Rhac084	-5.519722	104.45	
	85	Rhac085	-5.549754	104.439883	
Vietnam	86	Rhac086	11.264999	106.987652	iNaturalist, 2024
	87	Rhac087	11.3808	107.062	European Bioinformatics Institute (EMBL-EBI), GBIF Helpdesk (2025). INSDC Sequences. Version 1.140. European Nucleotide Archive (EMBL-EBI). Occurrence dataset https://doi.org/10.15468/sbmztx accessed via GBIF.org on 2025-06-10. https://www.gbif.org/occurrence/3346703935
	88	Rhac088	12.19	107.21	GBIF, 2024
	89	Rhac089	11.43	107.42	GBIF, 2024
	90	Rhac090	12.867	107.7	GBIF, 2024
	91	Rhac091	11.509481	108.154446	Borissenko, A.V. and Kruskop, S.V., BATS OF VIETNAM. 2003
	92	Rhac092	11.600594	105.899077	
	93	Rhac093	11.424168	107.423013	Thong, V.D., 2015. Bats of Cat Tien national park: diversity, echolocation and taxonomic remarks. Academia Journal of Biology, 37(3), pp.336-343.
	94	Rhac094	11.300743	107.066955	
	95	Rhac095	21.11951	104.977393	Görföl, T., Huang, J.C.C., Csorba, G., Győrössy, D., Estók, P., Kingston, T., Szabadi, K.L., McArthur, E., Senawi, J., Furey, N.M. and Tu, V.T., 2022. ChiroVox: a public library of bat calls. PeerJ, 10, p.e12445.
	96	Rhac096	16.611488	106.913891	
	97	Rhac097	11.162474	107.027232	Borissenko, A.V. and Kruskop, S.V., BATS OF VIETNAM.
Brunei	98	Rhac098	4.233	114.667	iNaturalist, 2024
Philippines	99	Rhac099	8.95	117.98333	Tanalgo K (2023). The MOBIOS+: A FAIR (Findable, Accessible, Interoperable, and Reusable) Database for Mindanao's Terrestrial Biodiversity. Biodiversity Data Journal. Occurrence dataset https://doi.org/10.15468/rtedgk accessed via GBIF.org on 2025-06-10. https://www.gbif.org/occurrence/4523543446

Supplementary: List of localities and data sources of *Rhinolophus acuminatus*

	100	Rhac100	9.8371	118.6413	Tanalgo K, Dela Cruz K (2025). BatMapPH: Philippine Bat Species Occurrence Database. Version 1.220. Eco/Con Lab Biodiversity Synthesis+ Centre. Occurrence dataset https://doi.org/10.15468/x8vxpr accessed via GBIF.org on 2025-06-10. https://www.gbif.org/occurrence/4906808835
	101	Rhac101	9.174361	124.716392	Bentley A, Krejsa D (2025). KUBI Mammalogy Collection. Version 26.85. University of Kansas Biodiversity Institute. Occurrence dataset https://doi.org/10.15468/a3woj7 accessed via GBIF.org on 2025-06-10. https://www.gbif.org/occurrence/686492451
	102	Rhac102	10.36275	118.986504	Grant S, Webbink K, Jones J, Ferguson A (2020). Field Museum of Natural History (Zoology) Mammal Collection. Version 9.18. Field Museum. Occurrence dataset https://doi.org/10.15468/n4zgxw accessed via GBIF.org on 2025-06-10. https://www.gbif.org/occurrence/665762335
	103	Rhac103	10.77397	119.533521	Esselstyn, J.A., Widmann, P. and Heaney, L.R., 2004. The mammals of Palawan island, Philippines. Proceedings of the Biological Society of Washington, 117(3), pp.271-302.
	104	Rhac104	9.996156	118.945063	
	105	Rhac105	10.05	119.0122	
	106	Rhac106	17.507794	121.937069	Cabauatan, J.G., Ramos, M.T., Taggueg, J.B., Callueng, A.M. and Tumaliuan, S.S., 2014. Assessment of faunal diversity on selected caves of the Northern Sierra Madre Natural Park (NSMNP), Northern Cagayan Valley, Philippines. *Journal of Agricultural Technology* 10(3):631-649
	107	Rhac107	9.531979	118.447617	Neil Jun Sala Lobite. 2017. Impacts of Lowland Rainforest Transformationto Rubber and Oil Palm on Multiple Dimensions of Chiropteran Diversity. [Master's thesis: University of Philippines Los Banos]
	108	Rhac108	10.779074	119.513948	Gonzales, B.J., Matillano, J.D. and City, P.P., 2014. Wetland Study of Lake Manguao as Special Co-management Area for Eco-tourism in the Province of Palawan.
Myanmar	109	Rhac109	14.7295	98.2469	GBIF, 2024
	110	Rhac110	12.453134	98.578345	Bates, P.J., Thi, M.M., Nwe, T., Bu, S.S.H., Mie, K.M., Nyo, N., Khaing, A.A., Aye, N.N., Oo, T. and Mackie, I., 2004. A review of Rhinolophus (Chiroptera: Rhinolophidae) from Myanmar, including three species new to the country. Acta Chiropterologica, 6(1), pp.23-48.
	111	Rhac111	17.761494	96.68653	
	112	Rhac112	17.455716	96.833016	
Thailand	113	Rhac113	6.937799	100.164167	Observation.org (2025). Observation.org, Nature data from around the World. Occurrence dataset https://doi.org/10.15468/5nilie accessed via GBIF.org on 2025-06-10. https://www.gbif.org/occurrence/5061314489
	114	Rhac114	14.01	101.71	iNaturalist contributors, iNaturalist (2025). iNaturalist Research-grade Observations. iNaturalist.org. Occurrence dataset https://doi.org/10.15468/ab3s5x accessed via GBIF.org on 2025-06-10. https://www.gbif.org/occurrence/5166794528
	115	Rhac115	9.042389	98.271636	Saelao T, Soisook P (2024). PSUZC-Mammal Collection. Princess Maha Chakri Sirindhorn Natural History Museum (PSU Museum, Prince of Songkla University, Thailand). Occurrence dataset https://doi.org/10.15468/xfkmp6 accessed via GBIF.org on 2025-06-10. https://www.gbif.org/occurrence/1830793227

	116	Rhac116	7.005816	100.499	Orrell T, Informatics and Data Science Center - Digital Stewardship (2025). NMNH Extant Specimen Records (USNM, US). Version 1.94. National Museum of Natural History, Smithsonian Institution. Occurrence dataset https://doi.org/10.15468/hnhrg3 accessed via GBIF.org on 2025-06-10. https://www.gbif.org/occurrence/1318624162
	117	Rhac117	6.93	100.549	Prestridge H (2024). Biodiversity Research and Teaching Collections - TCWC Vertebrates. Version 9.6. Texas A&M University Biodiversity Research and Teaching Collections. Occurrence dataset https://doi.org/10.15468/szomia accessed via GBIF.org on 2025-06-10. https://www.gbif.org/occurrence/675883946
	118	Rhac118	14.61	101.07	Conroy C (2025). MVZ Mammal Collection (Arctos). Version 35.95. Museum of Vertebrate Zoology. Occurrence dataset https://doi.org/10.15468/uwudf9 accessed via GBIF.org on 2025-06-10. https://www.gbif.org/occurrence/5147325572
	119	Rhac119	9.391897	98.792818	Qumsiyeh, M.B., Owen, R.D. and Chesser, R.K., 1988. Differential rates of genic and chromosomal evolution in bats of the family Rhinolophidae. Genome, 30(3), pp.326-335.
	120	Rhac120	9.10202	99.350971	
	121	Rhac121	14.59	101.9	Harada, M.; Minezawa, M.; Takada, S.; Yenbutra, S.; Nunpakdee, P.; Ohtani, S. Karyological Analysis of 12 Species of Bats from Thailand. *Caryologia* **1982**, *35*, 269–278, doi:10.1080/00087114.1982.10796932.
	122	Rhac122	9.18	98.58	Hood, C.S.; Schlitter, D.A.; Iliopoulou-Georgudaki, Joan.; Yenbutra, S.; Baker, R.J. Chromosomal Studies of Bats (Mammalia: Chiroptera) from Thailand. *Annals of the Carnegie Museum* **1988**, *57*, 99-109.
	123	Rhac123	9.06	99.01	
	124	Rhac124	13.158516	102.137	Kanphan, S.; Wongwai, A.; Soisuk, P. *Cave-Dwelling Bats of Thailand*; 1st ed.; Wildlife Research Division, Department of National Parks, Wildlife and Plant Conservation: Bangkok, Thailand, 2559; ISBN 978-616-316-356-1.
	125	Rhac125	14.88	102.14	SANBORN, C.C. The Mammals of the Rush Watkins Zoological Expedition to Siam. *Natural History Bulletin of the Siam Society* **1952**, *15*, 1–20.
	126	Rhac126	7.534	99.605	
	127	Rhac127	6.72	101.357	
	128	Rhac128	8.997619	98.460376	GBIF, 2024
	129	Rhac129	9.95	98.77	GBIF, 2024
	130	Rhac130	9.308644	98.946875	GBIF, 2024
	131	Rhac131	10.5	99.166672	GBIF, 2024
	132	Rhac132	15.06	100.1	GBIF, 2024
	133	Rhac133	5.5	101.4	GBIF, 2024
	134	Rhac134	5.795833	101.832222	GBIF, 2024
	135	Rhac135	5.806213	101.950819	GBIF, 2024
	136	Rhac136	13.4098451	101.880159	Wacharapluesadee, S.; Tan, C.W.; Maneeorn, P.; Duengkae, P.; Zhu, F.; Joyjinda, Y.; Kaewpom, T.; Chia, W.N.; Ampoot, W.; Lim, B.L.; et al. Evidence for SARS-CoV-2 Related Coronaviruses Circulating in Bats and Pangolins in Southeast Asia. *Nat Commun* **2021**, *12*, 972, doi:10.1038/s41467-021-21240-1.
	137	Rhac137	17.483792	98.4577126	Wildlife Yearbook Available online: https://www.dnp.go.th/wildlife/indexpageall.htm (accessed on 10 December 2022).
	138	Rhac138	9.290316	98.85336	
	139	Rhac139	7.950501	99.286902	

	140	Rhac140	15.479877	99.29269	
	141	Rhac141	15.693182	99.307501	
	142	Rhac142	13.304789	99.419697	
	143	Rhac143	8.592835	99.531702	
	144	Rhac144	7.188165	100.066931	
	145	Rhac145	5.885629	101.805249	
	146	Rhac146	9.092098	98.283839	Soisook, P., Karapan, S., Srikrachang, M., Dejtaradol, A., Nualcharoen, K., Bumrungsri, S., ... & Bogdanowicz, W. (2016). Hill forest dweller: a new cryptic species of Rhinolophus in the'pusillus group'(Chiroptera: Rhinolophidae) from Thailand and Lao PDR. Acta Chiropterologica, 18(1), 117-139.
	147	Rhac147	7.011509	100.513399	
	148	Rhac148	6.949893	100.231995	
	149	Rhac149	6.95008	100.232612	Phommexay, P., Satasook, C., Bates, P., Pearch, M. and Bumrungsri, S., 2011. The impact of rubber plantations on the diversity and activity of understorey insectivorous bats in southern Thailand. Biodiversity and Conservation, 20, pp.1441-1456.
	150	Rhac150	7.243939	100.410386	Boonchuay, P. and Bumrungsri, S., 2022. Bat activity in organic rice fields is higher than in conventional fields in landscapes of intermediate complexity. Diversity, 14(6), p.444.
	151	Rhac151	5.814851	101.844133	Samoh, A., Pantip, V. and Soisook, P., 2021. A checklist of Nycteribiid and Streblid Bat Flies (Diptera: Nycteribiidae and Streblidae) from Thailand with thirteen new records for the country. Tropical Natural History, 21(2), pp.244-262.
	152	Rhac152	7.624776	99.803121	Phommexay, P., Satasook, C., Bates, P., Pearch, M. and Bumrungsri, S., 2011. The impact of rubber plantations on the diversity and activity of understorey insectivorous bats in southern Thailand. Biodiversity and Conservation, 20, pp.1441-1456.
	153	Rhac153	14.493333	101.915982	Aroon, S., Hill III, J.G., Artchawakom, T., Pinmongkonkul, S. and Thanee, N., 2016. THE EFFECTS OF FOREST TYPE AND SEASON ON THE ABUNDANCE AND SPECIES DIVERSITY OF BATS IN NORTHEASTERN THAILAND. Suranaree Journal of Science & Technology, 23(3).
